# Two novel *FBN1* mutations associated with ectopia lentis and marfanoid habitus in two Chinese families

**Published:** 2009-04-23

**Authors:** Liming Zhao, Ting Liang, Jianzhen Xu, Hui Lin, Dandan Li, Yanhua Qi

**Affiliations:** 1 Department of Ophthalmology, Harbin Medical University the 2nd Affiliated Hospital, Harbin, China; 2 Center of Integrative Biology, Guangzhou Institute of Biomedicine and Health, Chinese Academy of Sciences, Guangzhou, China

## Abstract

**Purpose:**

To identify the molecular defects in the fibrillin-1 gene (*FBN1*) in two Chinese families with ectopia lentis (EL) and marfanoid habitus.

**Methods:**

Five patients and eight non-carriers in the two families underwent complete physical, ophthalmic, and cardiovascular examinations. Genomic DNA was extracted from leukocytes of venous blood of these individuals in the families as well as 100 healthy normal controls. Polymerase chain reaction (PCR) amplification and direct sequencing of all 65 coding exons of *FBN1* were analyzed. The functional consequences of the mutations were analyzed with various genomic resources.

**Results:**

Two novel mutations of *FBN1* were identified in our study. One is a splice defect in intron 17 (IVS 17–1G>T) adjacent to exon 18. The other is c.6182G>T in exon 50, which results in the substitution of cysteine by phenylalanine at codon 2,061 (p. C2061F). We provided strong evidences that the splice mutation would potentially lead to the skipping of exons after intron 17 and that the missense mutation at codon 2,061 (p. C2061F) would destroy a disulfide bond.

**Conclusions:**

We detected two novel mutations in *FBN1*. Our results expand the mutation spectrum of *FBN1* and help in the study of the molecular pathogenesis of Marfan syndrome and Marfan-related disorders.

## Introduction

Ectopia lentis (EL; OMIM 129600) is an inherited connective disorder characterized by lens dislocation, often connected with stretched or discontinuous zonular filaments [1]. In most cases, EL occurs as one symptom of Marfan syndrome (MFS; OMIM 154700), a genetic autosomal dominant disorder that is characterized by manifestations mainly involving the cardiovascular, skeletal, and ocular systems [2]. According to the Ghent nosology, a clinical diagnosis of MFS requires the involvement of all three systems with two major diagnostic manifestations [3]. Other disorders such as isolated EL or predominant EL with some skeletal features belong to Marfan-related disorders.

Both Marfan syndrome and Marfan-related disorders mainly result from mutations in the fibrillin-1 gene (*FBN1*) [4]. *FBN1* encodes a 320 kDa glycoprotein consisting of 2,871 amino acids and is located on chromosome 15q21. FBN1 is mainly composed of three types of repeated modules. The first one is the epidermal growth factor (EGF)-like module, which includes six cysteine residues. There are 47 such modules, and most of them are calcium binding (cb) EGF-like modules. The second type is called transforming growth factor β1-binding (or TB) protein-like module (TGF β1-BP-like module, or 8-Cys/TB), which is found seven times in FBN1. This module contains eight cysteine residues that form four disulfide bonds. The last one is a hybrid module, which occurs twice [5].

In this study, we analyzed two Chinese families with EL and marfanoid habitus and detected two novel heterozygous mutations in *FBN1* . In each family, the mutation found cosegregated in the patients and was not observed in any of the healthy family members.

## Methods

### Patients and clinical data

In our study, the patients from two Chinese families with ectopia lentis and marfanoid habitus were from the Heilongjiang province in northeastern China. Two patients and six non-carrier relatives in Family 1, three patients and two non-carrier relatives in Family 2, and 100 healthy normal controls were recruited for this study. The study was approved by the Institutional Review Board of Harbin Medical University (Harbin, China). After obtaining informed consent from all the participants, thorough physical, ophthalmic, and cardiovascular examinations were performed.

### Genomic DNA preparation

Blood specimens (5 ml) were collected in EDTA, and genomic DNA was extracted by the TIANamp Blood DNA Kit (Tiangen Biltech Co. Ltd, Beijing, China).

### Mutation screening

All coding exons of *FBN1* were amplified by polymerase chain reaction (PCR) using a set of 59 pairs of primers. The primers for exons 4, 5, 7, 11, 15, 22, 23, 31, 41, 44, 45, 51, and 52 were from those described by Li and coworkers [6]. The others are listed in Table 1. The PCR products were subsequently purified with a TIANgel Midi Purification Kit (Tiangen Biltech Co. Ltd) and sequenced with an ABI BigDye Terminator Cycle Sequencing kit v3.1 (ABI Applied Biosystems, Foster City, CA).

**Table 1:** Primers used for *FBN1* amplification.

**Exon**	**Forward primer (5′→3′)**	**Reverse primer (5′→3′)**	**Product length (bp)**
1	GGATTTGTCTCTGTGTTGCAG	CTTGCCAAGGAGTCTTCCAC	465
2	CTGCCAGGATTCATCTTGCT	AACTTTGACAGGGTTTGACCA	384
3	TTGTGAGGGACCTGAGAACC	TTTGGGCAGAACAGAGAAGG	340
6	TGCATGATTCTGTCCCTGAA	ATGCAGTCAGCGAAATTGTG	452
8	GCTGTTTCCAGGGACATGAT	AACCATGCATGCTGTTTGTC	267
9	GGGGCAGAGGTGTGAGTTAAT	CCCAAGTTTCCATTACATCTGC	380
10	TGACTTCTGTGGGCCTATGA	GAAGCCTCCCGTTTTTCTCT	448
12	GCTCAACCAGTCTTCAAATGG	CTTCCGGCATGGGTTATTTA	398
13	AATGGAGGGAGGGGGAAATA	AAATGGCAAGCTCTCCTAGC	420
14	ATGCACATGCCAAAACTCAA	TCCCAAACCAAAATTCAAGG	459
16	CAGAGGCATTCCCTGTGAGT	AAGACCCCAAGAAGGCACAT	407
17	TGATGTGTGCAAAACCAAGG	CATCCCAGATACATGGCACA	274
18	CCTCCTGTAGCTCCTAAGGTCA	AAGTGTCCATTTGCCCAGTC	348
19	CAGGAGTTTTGCCTTTTTGC	TGGCATTCCAAAAGATAGCA	308
20	AGCCCAGCTTTACTGTGTGG	TTTTGCAGGAAAAGCTGACA	313
21	AATGTCAGCTTTTCCTGCAA	CCCATTCAGCAATATGTTCG	437
24	GGCAAGGATACTTACCCCAGA	AAAGTCCATGCTGGGATGAT	506
25–26	AAAATGGTGGGCATTGAGAC	CCTCAGTCTCCCTCTGTTGC	601
27–28	AAGATGGACACCCAGCAATG	AGCGATGAAAACAAAACTCAGA	594
29	GATCCCACCATGAGGGTAGA	AAAGCCTGGGCCCTAAACTA	355
30	CCCAATGGGCTAGTTTATGC	GCTCTCTTTGGAATGCTGGT	444
32–33	TGGGAAGTTTGAAGGCAAGT	GCCTGAGAAATGTGGAATGC	566
34	TGCTGCACTGGAAAGTTGAT	GAAATGGTCAGCTGGAAACC	382
35	GAAGTGCCCAGATTGGTGTT	GTGACGGCCCTTGTGTAGTC	344
36	TCTCTGAAGTGGAAGACTGCAT	AGAATGGAATGTTTGGTGCTG	352
37	ACGGTTTTTGAACAGTTCCTG	ATTGGGAATAAGGTCCCCTCT	400
38–39	TCAGACGGGCAGAGTAACAA	CCTGGCTATGTTCGTGTTTAGA	556
40	GAGAGGACACGGATGAATGAA	AACAAGACAGTGAAGGGATGC	408
42	TTCTTTGCTGACCCCTATCC	ATTAGGTGGAGCTGCACAGG	303
43	GTCCCTATTGCCATCACCAC	TCCACACCATGCCCTTTACT	411
46	CCTGGTATCTTGCAGGGATG	CTGACTTCCTTTGCTGATGC	323
47	TGGCATTTCTTGTTTGGCTA	TTTTCCTCCAGGTTTCCAGA	372
48	GAAGTCATGCCAGTGGGAAC	CTTGCCAGAAGGATGAGACC	325
49	CCCTTTGTGTGTCCACATTG	CAGAGCTTTGCCATGTTTGA	295
50	ATTGCTGTGGTCCTGAGAGG	TTACATCATGGCCAGTCTGC	329
53	AGCACTGCAGTCTGGATGTC	AATGATCAAATGGCCCATCA	399
54	GGAAATGGGAGACCACTTGA	ATTCCAATTCCCAGCCTTCT	371
55	GCAGAAGGAAATACAGCCAGT	GGGTCTCGCCAAGAACAGTA	372
56	GAACAAAGGGAGGGAAGGAG	CAGTCATTACGGCATCTCCA	389
57	GCTTTCCCCTCTTGCTTCTT	GGCACATATTGCAACTCCA	441
58–59	CACTGAAGTGACCCCCTACAT	AAGCACCTCCTGCCTGTAGA	688
60	AATCAAACGTGGAGCTGCTT	AAAGGCCAAATAAGGCCAAC	382
61	AGCGTTGTTGGCCTTATTTG	CCTGGGCTCAGATCTGCTAT	356
62	TAGGATGTGTAGGGGCCAGA	TTCAACCAGGTTAGGGCAAT	349
63	AGCCACCTCTGCCTGTCTTA	AAAGCATGGTTCTCCTCTGC	473
64	TCACAACTGCAAGGAACAGG	ACACTTTGGAGCATCCTTGG	362
65	GCAGCATAAGGCAGAAAATTG	TCACCTGTACCTTGCTTTGG	667

Summary of the primers used for the amplification of *FBN1* exons. Sequences are given in the 5′→3′direction.

### Information theory mutational analysis

The potential results of the G→T transversion were estimated using information theory as described in the literature [7]. Briefly, potential splice sites were identified by the splice mutation analysis system based on information theory. Thus, the score of the site containing a mutant nucleotide would be significantly changed compared with that of the wild-type splice site. The analysis had been previously used for the interpretation of other mutations [8,9]. We used walker [10] visualization maps to present the predicted changes in binding sites.

### Structure analysis

The protein structure file, 1apj, downloaded from the Protein data bank (PDB) database, demonstrates the solution structure of the transforming growth factor beta binding (TB) protein-like domain 6 of fibrillin (residues 2054–2125) [11]. This structure was displayed with the KiNG viewer to show the missense mutation at codon 2061.

## Results

### Clinical findings

**Figure 1:**
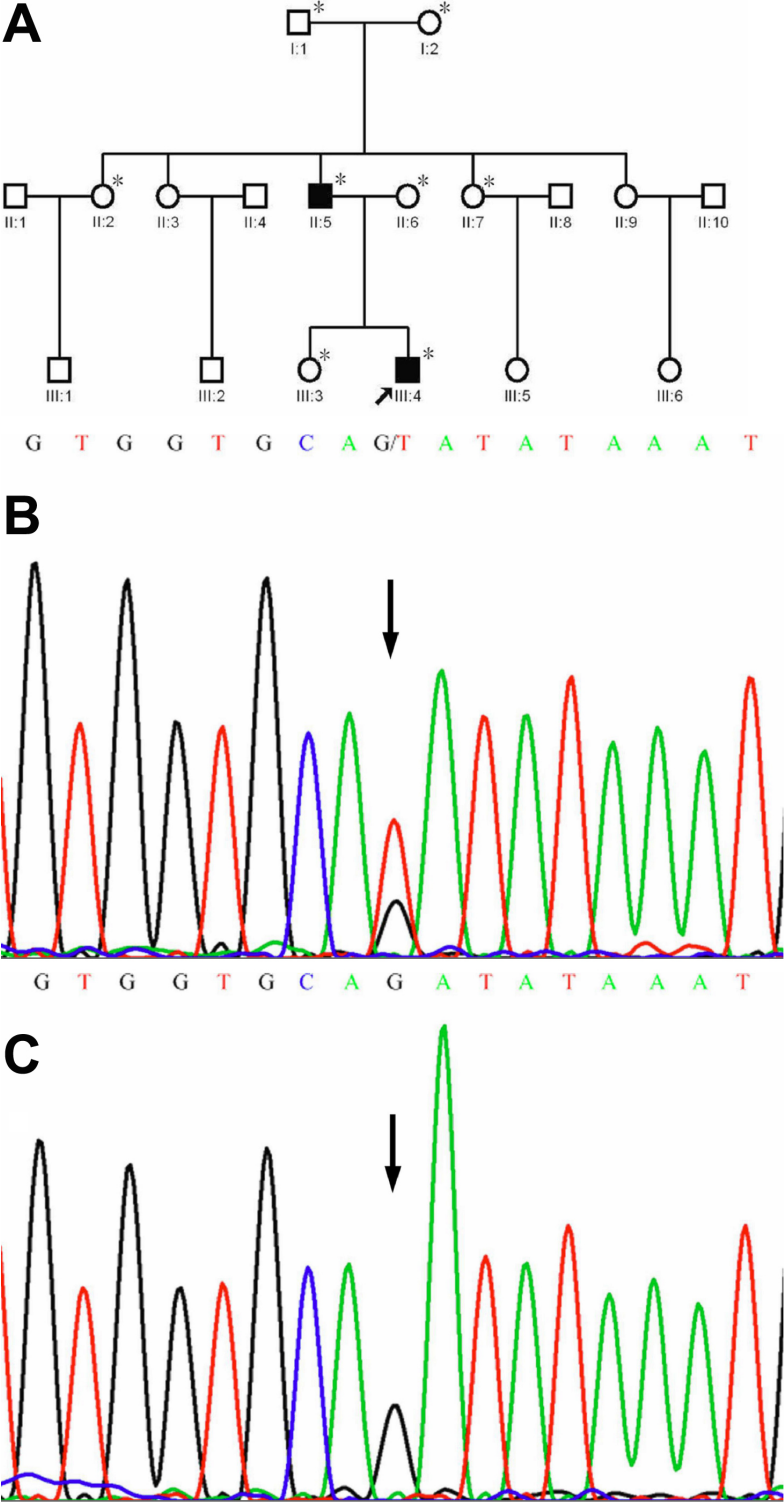
A novel *FBN1* splice mutation in intron 17. **A**: The pedigree of Family 1 is shown. Squares and circles indicate males and females, respectively, and the darkened symbols represent the affected members. The patient above the arrow is the proband. An asterisk indicates the subject underwent clinical and molecular analyses. **B**: The partial nucleotide sequence of *FBN1* in an affected member is shown. A heterozygous change G>T (indicated by the arrow) was identified at the boundary of intron 17 and exon 18. **C**: The corresponding normal sequence in an unaffected family member is displayed by an arrow.

**Figure 2:**
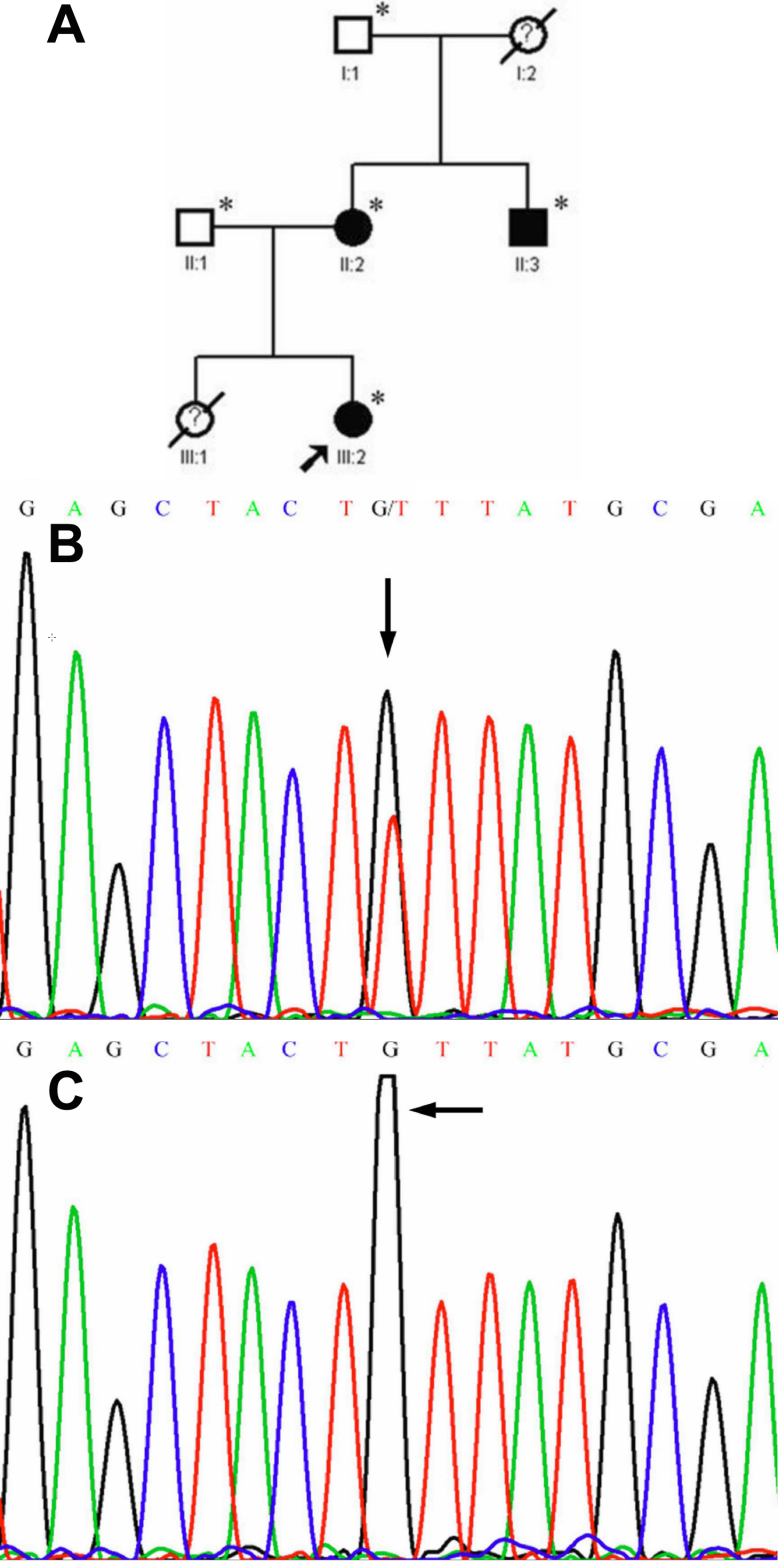
A novel *FBN1* missense mutation in exon 50. **A**: The pedigree of Family 2 is shown. Slashed symbols denote that the subject is deceased. Symbols with a question mark in the center indicate that the member is not diagnosed clearly. **B**: A heterozygous G>T transversion (indicated by the arrow) resulted in the substitution of cysteine-2061 by phenylalanine (C2061F) in an affected subject. **C**: The corresponding normal sequence in an unaffected family member is shown by an arrow.

In the two families, all the patients (Figure 1A, Figure 2A) in our study showed similar clinical symptoms (Table 2). Bilateral lens dislocation was discovered in the five patients, and none of them displayed any abnormalities in the cardiovascular system by echocardiogram. However, in Family 2, individual I:2 died of congenital heart disease at the age of 30 years old with big hands according to the description of her daughter (II:2), and her granddaughter (III:1) also died of congenital heart disease only four days after birth. It was not clear whether they had any other abnormalities such as EL because they were deceased several years ago and no related medical records were available. As for the skeletal system, arachnodactyly was present in the five patients.

**Table 2:** Clinical details of the five patients from the two families.

**Manifestation**	**Family 1**		**Family 2**		
**Patient**	**II:5**	**III:4**	**II:2**	**II:3**	**III:2**
Age (Years)	39	11	24	20	4
Sex	M	M	F	M	F
**Ocular system**					
Ectopia lentis	+	+	+	+	+
Myopia	+	+	+	+	+
Abnormally flat cornea	–	–	–	–	–
Early development of cataract	–	–	–	–	–
Strabismus	+	+	+	+	+
Glaucoma	–	–	–	–	–
Retina detachment	–	–	–	–	–
**Skeletal system**					
Height (cm)	193	153	168	174	107
Arm span (cm)	194	151	171	172	107
AS/H (normal<1.05)	1.01	0.99	1.02	0.99	1
Pectus carinatum	–	–	–	–	–
Pectus excavatum	–	–	–	–	–
Scoliosis	–	–	–	–	–
Arachnodactyly	+	+	+	+	+
High palate with dental crowding	–	–	–	–	–
Joint hypermobility	–	–	–	–	–
Flatfoot	–	–	–	–	–
**Other manifestations**					
Hyperextensible skin	–	–	–	–	–
Hernia	–	–	–	–	–

Affected family members had bilateral ectopia lentis, and arachnodactyly. Abnormalities in the cardiovascular system were absent. Abbreviations: M: male; F: female; AS/H: arm span/height ratio; +: present, –: absent.

### Mutation analysis

After direct sequencing of *FBN1* in the five patients, a splice defect in intron 17 (IVS 17-1G>T) adjacent to exon 18 (Figure 1B) and a missense mutation involving the substitution of cysteine by phenylalanine in exon 50 (p.C2061F; Figure 2B) were discovered in Family 1 and Family 2, respectively. Neither of the two mutations was detected in the healthy family members (Figure 1C, Figure 2C) or any of the 100 unrelated control subjects.

### Potential functional consequences of the two mutations

**Figure 3:**
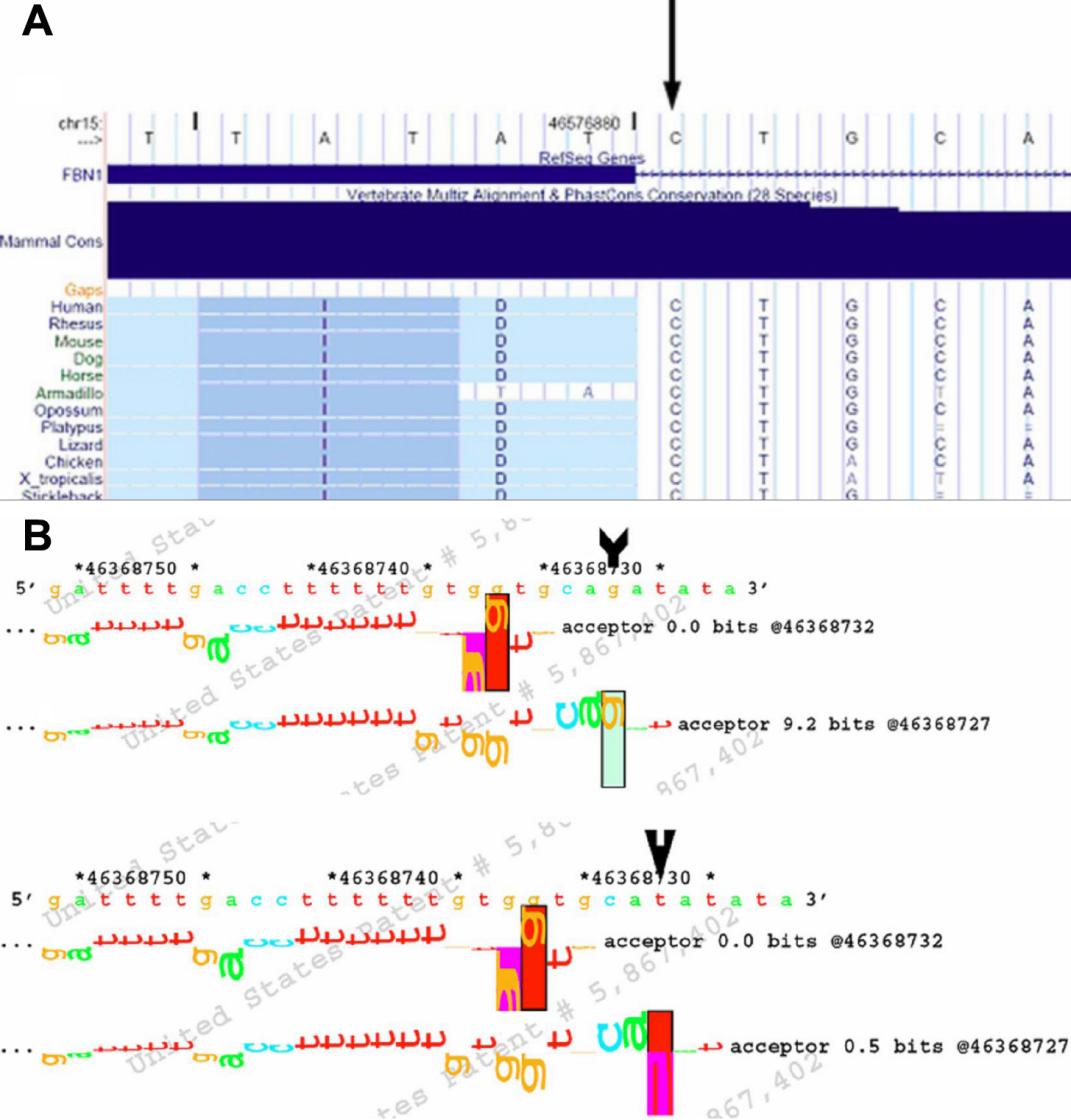
Analysis of the splice mutaion in intron 17. **A**: The alignment of the FBN1 sequence with the corresponding segments in diverse species is displayed. The nucleotide G is conserved in FBN1 proteins from several species. The sequence was selected by UCSC Genome Browser. Note that *FBN1* is located at the minus strand, and the nucleotide sequence of this genomic region is represented by the plus strand. The 'Mammal Cons’ is a conservation measurement. **B**: The walker diagram of 3′ (acceptor) splice site in intron 17 and its adjacent sequence is shown. The wild-type sequence is at the top. Bases in splice sites are shown in the corresponding walker diagram. The arrow points to the mutant sequence with the G to T base change and shows the change from a positive contribution by the G to a near zero contribution of the T.

**Figure 4:**
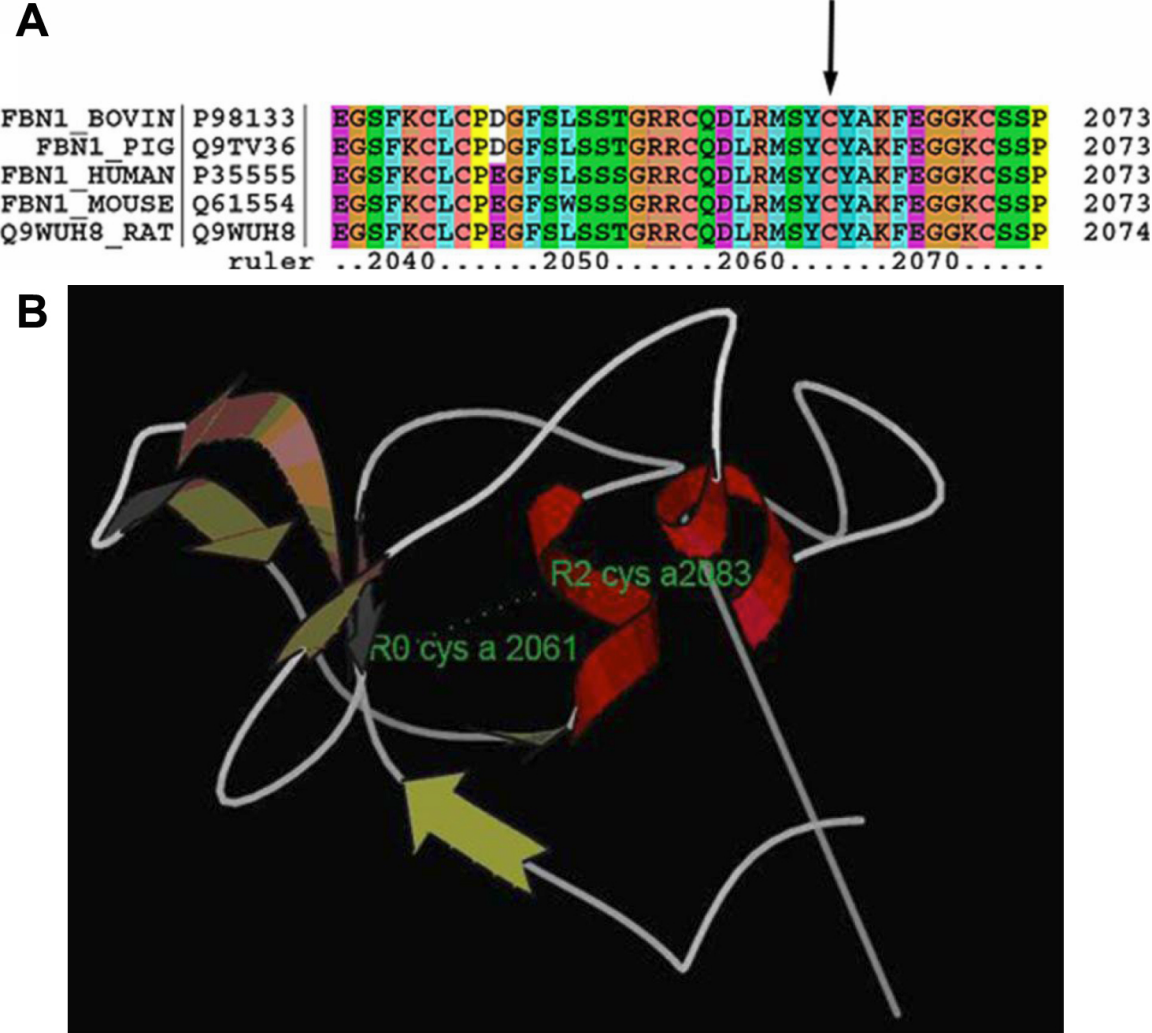
Analysis of the missense mutation in exon 50. **A**: The alignment of the FBN1 sequence with the corresponding segments in diverse species is shown. The cysteine is conserved in FBN1 proteins from several species. The sequence was selected from the UniProt Knowledge base. **B**: Structure analysis of the transforming growth factor-binding protein-like domains (8-Cys/TB) in human FBN1. α-helices and β-strands are shown with red and brown colors. The two residues (C2061 and C2083) are colored green. The disulfide bond is represented with a dotted line.

The IVS 17–1G>T mutation located at a highly conserved splice site of intron 17, which has canonical GT/AG ends (Figure 3A). Information theory analysis revealed that the information contents (Ri) value decreased from 9.2 bits to 0.5 bits by the mutation (Figure 3B). The cysteine residue at position 2,061 was also conserved among mammalian species (Figure 4A). Structure analysis of the transforming growth factor β (TGF-β)-binding protein-like domain revealed that C2061 and C2083 formed one of the four disulfide bonds. (Figure 4B) [11].

## Discussion

In this study, we described two novel heterozygous mutations in *FBN1* (IVS 17–1G>T and p.C2061F). Furthermore, we used various genomic resources to analyze the potential functional consequences of these two mutations.

In Family 1, it was a splice mutation in position 1 of the intron 17-exon 18 boundary in the domain of cb EGF-like number 07. EGF-like domains play a major role in the pathogenesis of fibrillinopathies containing 75% of all the *FBN1* mutations registered in the FBN1 Universal Mutation Database (UMD) database. Previously, Rogan et al. [12] showed that the minimum Ri value for a functional splice site was 2.4 in a study of over 100 splice sites. As for the splice mutation in our study, the Ri value decreased from 9.2 bits to 0.5 bits. The mutation of this base would be expected to disrupt the acceptor site and potentially lead to abnormal mRNA splicing and skipping of exons after intron 17. This also supports the observation that splice mutations often lead to a shortened protein, accounting for about 11%–12% of the gene lesions in *FBN1* [5,13]. Interestingly, the c. 2168–1G>T splice site mutation (in IVS 17) involved the same nucleotide of the c. 2168–1G>A substitution previously described in *FBN1* [14].

Family 2 carried a missense mutation affecting cysteine residues in exon 50 in the domain of 8-Cys/TB number 06. This supports the previous studies that mutations involving cysteine substitution are usually associated with EL [13,15,16]. Each 8-Cys/TB module contains eight highly conserved cysteine residues holding TGF-β in an inactive complex in various tissues including the extracellular matrix [17]. Structure analysis showed C2061 and C2083 form one of the four disulfide bonds. Therefore, the substitution of cysteine by phenylalanine in this position was likely to destroy the disulfide bond and cause domain misfolding and structure instability. Recent studies demonstrated that increased TGF-β signaling contributed to selected symptoms of MFS [18] and could cause dysregulation of cytokine function in mouse models of MFS [19]. All above show that 8-Cys/TB domains also play an important role in the pathogenesis of fibrillinopathies.

Since *FBN1* cDNA was cloned and the first mutations of *FBN1* were identified in MFS patients in 1991 [20-22], currently more than 1,200 *FBN1* mutations have been described [23]. Most of them are missense mutations, and others are nonsense mutations, splice defect, deletions, and so on. In this study, we described two novel heterozygous mutations in *FBN1*  in the Chinese patients with ectopic lentis and marfanoid habitus and analyzed the potential functional consequences of the two mutations. Our data further expand the mutation spectrum of *FBN1* and help in the study of molecular pathogenesis of Marfan syndrome and Marfan-related disorders.
